# Proprioceptive Rehabilitation Prevents Ankle Sprain Following L5-S1 Radiculopathy: A Case Report

**DOI:** 10.7759/cureus.89440

**Published:** 2025-08-05

**Authors:** Mohamed Harmouche, Sarra El Hamlili, Siham Elmir, Younes El Anbari, Ahmed Amine El Oumri

**Affiliations:** 1 Physical Medicine and Rehabilitation, Faculty of Medicine and Pharmacy, Mohammed I University, Oujda, MAR; 2 Physical Medicine and Rehabilitation, Mohammed VI University Hospital Center, Oujda, MAR; 3 Physical Medicine and Rehabilitation, Faculty of Medicine and Pharmacy, Sultan Moulay Slimane University, Beni-Mellal, MAR; 4 Physical Medicine and Rehabilitation, Beni-Mellal University Hospital Center, Beni-Mellal, MAR

**Keywords:** ankle instability, ankle sprain, faam score, l5-s1 radiculopathy, neuromuscular rehabilitation, peripheral neuropathy, primary prevention, proprioceptive rehabilitation, star excursion test, unipodal balance

## Abstract

This case report describes the functional outcomes of a proprioceptive rehabilitation protocol for primary prevention in a 31-year-old patient with L5-S1 radiculopathy sequelae confirmed by electroneuromyography and associated ankle proprioceptive deficit. The patient underwent a seven-week proprioceptive rehabilitation protocol comprising 10 supervised sessions, with a six-month follow-up period. Assessment tools included the Foot and Ankle Ability Measure (FAAM), the single-leg balance test with eyes closed, and the visual analog scale (VAS) for perceived stability. At six-month follow-up, significant improvements were observed: FAAM activities of daily living (FAAM-ADL) score increased from 68% to 94%, FAAM sports activities (FAAM-Sport) score improved from 45% to 87%, single-leg balance time with eyes closed extended from 3 to 18 seconds, and VAS for perceived stability increased from 3/10 to 8/10. No ankle sprain episodes were reported during the follow-up period. These findings suggest that preventive proprioceptive rehabilitation effectively reduces ankle sprain risk in patients with peripheral neurological deficits. This primary prevention approach demonstrates promising results for managing functional deficits secondary to radiculopathy and warrants evaluation in larger cohorts. The protocol's success highlights the importance of targeted proprioceptive training in preventing musculoskeletal complications in neurologically compromised patients.

## Introduction

Lumbar radiculopathies affect 3-5% of the adult population, with L5-S1 nerve root involvement accounting for nearly 90% of cases [1,2]. This neurological compromise specifically impairs the innervation of ankle-stabilizing muscles, including the peroneal muscles and triceps surae, creating a periarticular muscular imbalance that significantly increases the risk of ankle instability and sprains [2,3]. While no specific studies have quantified the risk of ankle sprains in patients with L5-S1 radiculopathy, evidence from similar peripheral neuropathic conditions provides relevant insights. Patients with diabetic peripheral neuropathy demonstrate a 2.4-fold increased risk of developing ankle sprains compared to the general population, attributed to altered dynamic postural control mechanisms resulting from proprioceptive dysfunction and distal muscle weakness [4]. Given the similar patterns of sensory and motor deficits in L5-S1 radiculopathy affecting ankle-stabilizing muscles, comparable risks may exist, though specific epidemiological data for this population are lacking.

While proprioceptive rehabilitation is well-established for secondary prevention after initial ankle sprain, with meta-analyses showing a 38% reduction in recurrence risk [5], its efficacy remains insufficiently documented for primary prevention in patients with peripheral neuropathy before any sprain episode [6]. This gap in the literature is concerning given that this population presents major intrinsic risk factors that could benefit from targeted preventive interventions. This case report examines the functional improvements and clinical outcomes following a preventive proprioceptive rehabilitation protocol in a patient with documented L5-S1 radiculopathy sequelae, utilizing validated assessment tools including the Foot and Ankle Ability Measure (FAAM) score and standardized balance tests over a six-month follow-up period.

## Case presentation

A 31-year-old male engineer who frequently travels on uneven terrain for work presented with a six-month history of progressive difficulty walking on his heels and left ankle instability. These symptoms significantly impaired his professional activities, with multiple near-fall episodes without actual trauma. The patient had consulted several physicians who prescribed ankle radiographs (showing no bone abnormalities), rest periods, and 15 days of ankle bracing, all without clinical improvement, prompting specialized physical medicine consultation.

The patient reported a one-year history of L5-S1 left lumbosciatica, initially managed with physical therapy and anti-inflammatory treatment. While pain partially improved, distal neurological deficit persisted. Ankle instability developed progressively six months after radiculopathy onset, worsening over the last three months. Lumbar magnetic resonance imaging performed eight months prior revealed a left paramedian L5-S1 disc herniation contacting the S1 root without severe compression signs or surgical indication per neurosurgical consultation. The patient had no significant medical comorbidities, including diabetes mellitus, cardiovascular disease, or other neurological disorders. He was not taking any regular medications at the time of evaluation, with NSAIDs being discontinued three months prior. He had no history of previous surgeries, particularly spinal or lower extremity procedures. The patient's body mass index was 23.5 kg/m². He had regularly hiked before symptom onset and had no history of ankle sprains or familial ligamentous pathology.

Physical examination revealed visible amyotrophy of the left extensor digitorum brevis and subtle tibialis anterior atrophy. The muscle wasting was graded as moderate for the extensor digitorum brevis (visible depression in the dorsal foot) and mild for the tibialis anterior (asymmetry noticeable on comparative inspection). Manual muscle testing using the Medical Research Council scale showed left ankle dorsiflexor strength at 4/5, extensor hallucis longus at 3/5, peroneal muscles at 4/5, and triceps surae at 5/5, with abnormal fatigability on repeated testing. Neurological examination demonstrated absent left Achilles reflex contrasting, with present and symmetric patellar reflexes. Superficial sensation testing revealed hypoesthesia in the lateral L5 territory extending to the dorsum of the foot. Ankle ligamentous tests including anterior drawer and forced varus were negative bilaterally, excluding associated mechanical instability. The absence of generalized hyperlaxity (Beighton score 2/9), joint inflammatory signs, and recent trauma excluded hypermobility syndrome, arthritis, and post-traumatic instability, respectively.

Standardized functional assessment using the FAAM revealed the initial FAAM-Activities of Daily Living (FAAM-ADL) score of 68% (57/84 points), indicating moderate limitation in daily activities. The FAAM-Sport activities (FAAM-Sport) score was more severely affected at 45% (13/29 points), reflecting major apprehension during physical activities. Balance testing showed marked deficits with single-leg stance time eyes closed limited to 3 seconds on the affected side versus 32 seconds on the healthy side. With eyes open, the patient maintained balance for 22 seconds left versus 68 seconds right. Visual analog scale (VAS) assessing perceived stability was rated 3/10, indicating significant instability sensation. Star Excursion Balance Test evaluating dynamic balance showed an 18% reach deficit on the left side compared to the contralateral side.

**Figure 1:**
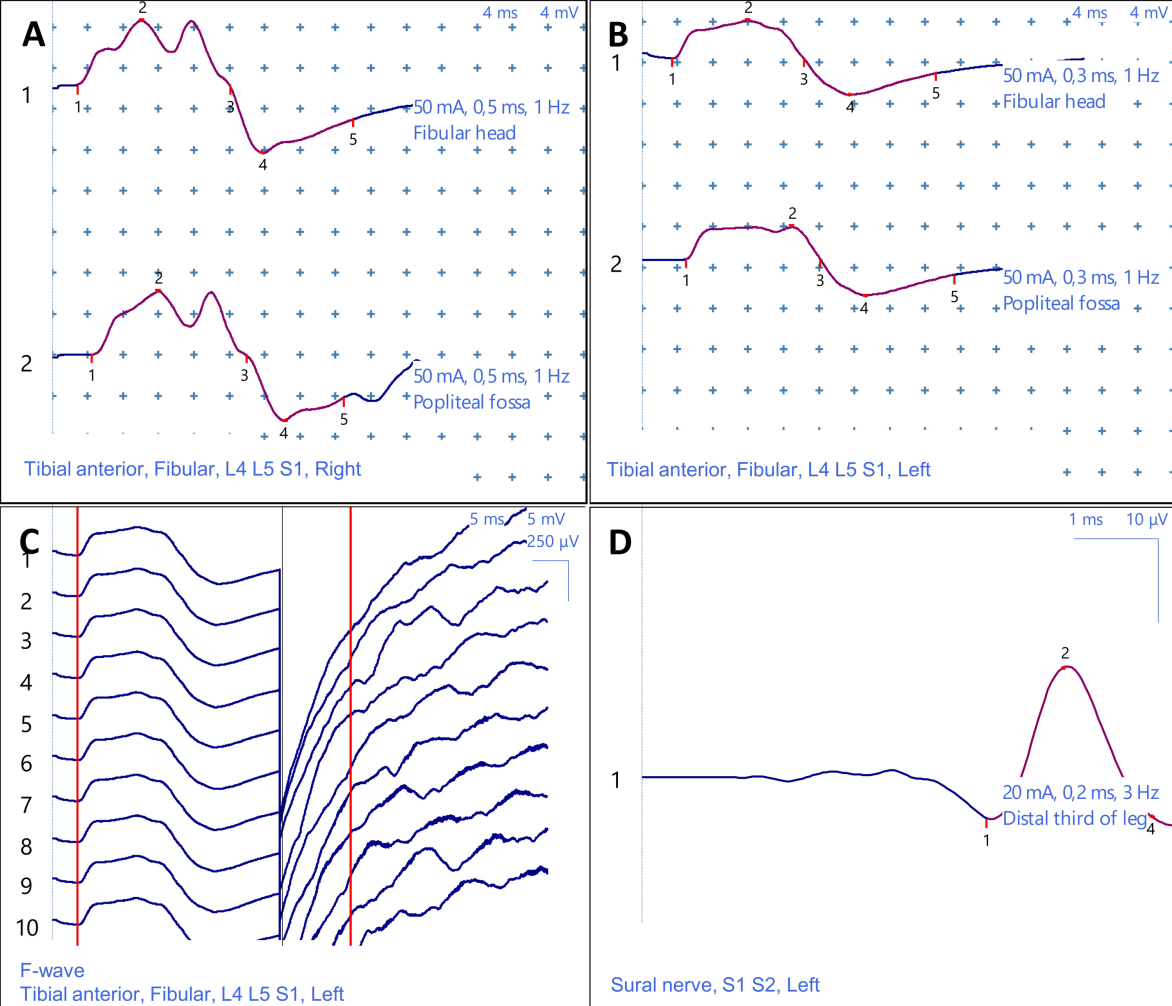
Electroneuromyographic findings in chronic L5 radiculopathy demonstrating asymmetric axonal loss (A) Motor nerve conduction study of the right fibular nerve showing normal compound muscle action potential amplitude at 6.3 mV and conduction velocity of 50.1 m/s. (B) Motor nerve conduction study of the left fibular nerve demonstrating a markedly reduced compound muscle action potential amplitude at 3.7 mV (41% reduction compared to the contralateral side) and preserved conduction velocity at 53.6 m/s, consistent with axonal loss. (C) The F-wave study of the left fibular nerve showing prolonged minimal latency at 42.0 ms, indicating proximal nerve involvement. (D) Sensory nerve conduction study of the left sural nerve showing preserved amplitude at 18.1 µV and normal conduction velocity at 43.7 m/s, excluding distal peripheral neuropathy.

Electroneuromyography (EMG) confirmed chronic peripheral neurological involvement. Motor conduction studies showed significant reduction in left fibular nerve compound muscle action potential amplitude at 3.7 mV (Figure 1B) versus 6.3 mV contralaterally (Figure 1A), with conduction velocity slowing to 53.6 m/s. Sensory conduction demonstrated moderate slowing of the left musculocutaneous nerve at 43.0 m/s, while sural nerve conduction remained preserved at 43.7 m/s (Figure 1D). The F-wave study showed prolonged minimal latency at 42.0 ms (Figure 1C). Needle examination revealed chronic partial denervation signs in L5 and S1 territories, with rare fibrillation potentials and reinnervation motor unit potentials in tibialis anterior and medial gastrocnemius.

The proprioceptive rehabilitation protocol (Table 1) comprised two supervised phases supplemented by a home exercise program. Phase 1 (six sessions over three weeks) focused on proprioceptive restoration through progressive static work on unstable surfaces and stabilometry platform with visual feedback. Phase 2 (four sessions over four weeks) integrated dynamic exercises reproducing the patient's occupational demands. No significant adverse effects were reported except transient muscle soreness. The patient completed 10/10 supervised sessions (100% adherence) and reported 90% compliance with the home program.

**Table 1:** Progressive proprioceptive rehabilitation protocol Note: No specific licenses were obtained for the assessment tools used in this study. All tools were employed as part of standard clinical practice at our institution. FAAM-ADL, Foot and Ankle Ability Measure activities of daily living; ROM, range of motion

Phase	Duration	Intervention Type	Specific Exercises	Exercise Parameters	Progression Criteria
Phase 1: static	Three weeks (six sessions)	Surface progression	Stable → foam → Freeman → balance board → stabilometry	3 sets × 10 repetitions, 30 seconds hold, 30 seconds recovery	Single-leg stance >10 seconds
		Targeted strengthening	Tibialis anterior, peroneals, extensor hallucis	3 sets × 12 repetitions, 2 seconds isometric hold, 30 seconds between sets	Pain-free execution
		Sensory stimulation	Plantar sensory exercises, textured surfaces	2 sessions × 5 minutes, continuous stimulation	Enhanced perception
Phase 2: dynamic	Four weeks (four sessions)	Balance challenges	Stabilometry with visual feedback	3 sets × 8 repetitions, 45 seconds activity, 45 seconds recovery	FAAM-ADL >85%
		Functional training	Obstacle courses, direction changes	3 sets × 5 passages, 1 minute recovery between sets	Movement confidence
		Eccentric strengthening	Heel drops, resisted dorsiflexion	3 sets × 10 repetitions, 3 seconds eccentric phase, 45 seconds recovery	Full ROM achieved
Home program	Continuous	Maintenance	Single-leg balance, heel raises	2 sets × 12 repetitions daily, 20 seconds hold	>80% compliance
		Prevention	Proprioceptive exercises before sports	1 set × 10 repetitions, pre-activity warm-up	No ankle sprains

Additionally, a class 2 proprioceptive ankle brace was prescribed for unstable terrain activities, providing augmented sensory feedback without mobility restriction. A 15-minute daily home exercise program was taught, including single-leg balance exercises, intrinsic foot muscle strengthening, and posterior chain stretching. Ergonomic counseling included work surface height adjustment, use of shoes with reinforced heel counters, and temporary avoidance of pivot sports during rehabilitation.

The six-week evaluation showed significant functional improvement with FAAM-ADL score increasing to 93% (78/84 points) and FAAM-Sport progressing to 76% (22/29 points). Single-leg stance time eyes closed quadrupled to 12 seconds, and perceived stability VAS improved from 3/10 at baseline to 7/10. The patient reported restored confidence in daily movements with apprehension resolution.

At three months, functional gains consolidated with FAAM-ADL maintained at 94% (79/84 points) and FAAM-Sport continuing progression to 83% (24/29 points). Single-leg stance eyes closed reached 15 seconds and perceived stability stabilized at 8/10. Star Excursion Balance Test showed only 9% residual deficit. The patient had progressively resumed jogging on flat terrain without instability episodes.

The six-month final evaluation confirmed durable maintenance of gains. FAAM-ADL remained stable at 94% (79/84 points), indicating near-complete functional recovery for daily activities. FAAM-Sport continued improving to 87% (25/29 points). Single-leg stance time eyes closed reached 18 seconds, representing 500% improvement from baseline, and 48 seconds eyes open. Star Excursion Balance Test showed a minimal 7% deficit (Table 2). No ankle sprains or near-falls occurred during the six-month follow-up. While the initial improvements (zero to three months) can be primarily attributed to the proprioceptive protocol, the continued gains from three to six months likely reflect a combination of the maintained home exercises and the patient's progressive return to jogging, which began at month 3. The patient resumed all professional activities including uneven terrain travel without apprehension or systematic brace use. He maintained his home program at three 10-minute sessions per week integrated into his morning routine alongside his self-initiated running activities.

**Table 2:** Evolution of functional assessment parameters from baseline to six months post-intervention Assessment tools: The FAAM, VAS, and Star Excursion Balance Test were used as part of routine clinical assessment. No specific licenses were required or obtained for these standard clinical tools at our institution. FAAM-ADL, Foot and Ankle Ability Measure-Activities of Daily Living; FAAM-Sport: Foot and Ankle Ability Measure-Sports activities; VAS stability, visual analog scale for perceived stability

Parameter	Baseline	6 weeks	3 months	6 months	Total Change
FAAM-ADL (%)	68	93	94	94	+26 points
FAAM-Sport (%)	45	76	83	87	+42 points
Single-leg stance eyes closed (sec)	3	12	15	18	+15 sec
Single-leg stance eyes open (sec)	22	38	42	48	+26 sec
VAS stability (/10)	3	7	8	8	+5 points
Star Excursion Balance Test (deficit %)	18	12	9	7	-11 points

## Discussion

This case illustrates the functional benefits of a proprioceptive training approach as primary prevention in a patient with L5-S1 radiculopathy sequelae and distal neurological deficit. The observed functional improvement demonstrated substantial gains, with a FAAM-ADL progression of 26 points (from 68% to 94%) and FAAM-Sport of 42 points (from 45% to 87%). While these improvements appear clinically meaningful, specific minimal clinically important difference values for radiculopathy patients have not been established in the literature, making direct comparisons with thresholds from other pathologies difficult [7].

L5-S1 radiculopathy induces a complex pathophysiological cascade compromising distal joint stability [1,2]. The direct motor deficit primarily affects muscles innervated by L5 (tibialis anterior, extensor hallucis longus, peroneal muscles) and S1 (gastrocnemius, soleus) [1], creating periarticular force imbalances favoring forced inversion movements, the primary injury mechanism for sprains [3]. Recent electrophysiological studies have demonstrated that this weakness is accompanied by altered spinal reflex excitability [8], with compensatory bilateral inhibition suggesting central reorganization of motor control circuits [3].

Proprioceptive afferent alteration constitutes the second major mechanism of sprains [6]. Radicular compression induces segmental demyelination, preferentially affecting large-caliber myelinated fibers responsible for proprioceptive transmission [1]. This degradation compromises feedback mechanisms necessary for postural adjustments, increasing vulnerability to perturbations [3]. Ahmad et al. demonstrated that this alteration in peripheral neuropathies could be significantly improved through targeted sensorimotor training [4,9].

The absent Achilles reflex in our patient reflects S1 myotatic reflex arc interruption and deprives the nervous system of a rapid protective mechanism, increasing traumatic and sprain risk [3]. The originality of our approach lies in proprioceptive intervention before the occurrence of a first sprain episode. A recent meta-analysis by de Vasconcelos et al. established proprioceptive rehabilitation effectiveness with a 38% sprain risk reduction in athletic populations [5]. Our case suggests comparable efficacy of this approach in primary prevention among neurological patients.

The protocol used integrates evidence-based recommendations adapted to neurological deficit specificities [6]. The six-week duration with tri-weekly frequency corresponds to optimal parameters identified in Su et al.'s meta-analysis, recommending a minimum of six weeks with three sessions of 20 to 30 minutes [10]. Progression from static to dynamic work and functional exercise integration follows essential neuroplasticity principles [10].

Observed improvements are consistent with results reported in comparable populations. Studies on diabetic neuropathies show that single-leg balance improvements of 300% to 600% after proprioceptive training [4,9]. The 500% improvement observed in our patient falls within this range. Star Excursion Balance Test evolution, from an 18% deficit to 7%, is comparable to gains reported by McKeon et al. in patients with chronic instability [11]. The persistence of minimal residual deficit might reflect permanent neurological sequelae.

Mechanisms underlying improvement involve multiple nervous system levels. At the peripheral level, proprioceptive training optimizes residual afferent function through increased muscle spindle sensitivity and might promote partial axonal regeneration [12,13]. At the spinal level, rehabilitation modulates reflex circuit excitability, partially restoring feedback loops [8]. Lasting modifications of motoneuron excitability have been demonstrated after repeated training [14]. At the supraspinal level, training induces cortical reorganizations with increased somatosensory representation and improved sensorimotor integration [6,15]. The sustained improvements from three to six months likely reflect a synergistic effect between our proprioceptive protocol and the patient's progressive return to jogging initiated at month 3, highlighting the complementary benefits of structured rehabilitation and gradual activity resumption.

This observation underscores the importance of systematic proprioceptive deficit screening in patients with L5-S1 radiculopathy sequelae. Using validated tools such as FAAM allows objective quantification and standardized follow-up [7]. The timed single-leg balance test constitutes an excellent screening tool [16]. Early intervention, before sprain occurrence, appears particularly relevant in this at-risk population. The modest cost of a preventive program contrasts with potential consequences of repeated sprains [17].

Practical recommendations include: systematic screening within three to six months following diagnosis, particularly in active patients; inclusion criteria comprising motor deficit greater than or equal to 3/5, absent Achilles reflexes, single-leg balance less than 10 seconds, or at-risk activities; and quarterly follow-up during the first year with self-maintenance program. Integrating this approach into standardized pathways could optimize functional outcomes and reduce complications.

This observation presents limitations inherent to single cases. The absence of a control group does not allow exclusion of spontaneous improvement, although symptom chronicity and temporal correlation with intervention make this hypothesis unlikely. Proprioceptive assessment would have gained precision with a force platform. The absence of EMG reassessment at six months is explained by documented dissociation between functional recovery and electrophysiological normalization in chronic radiculopathies [18,19], making significant modifications unlikely to be observed. This decision aligned with the patient's wish to avoid an uncomfortable examination without expected therapeutic benefit.

The strengths of this study include validated tool utilization, six-month follow-up, electrophysiological documentation confirming diagnosis, and reproducible protocol description. These promising results warrant randomized controlled studies to validate this approach. Medico-economic evaluation including direct costs and potential savings would provide arguments for implementation. Developing protocols using new technologies could improve long-term compliance.

## Conclusions

This case report demonstrates that a seven-week proprioceptive rehabilitation protocol resulted in significant functional improvements in a patient with L5-S1 radiculopathy sequelae, including a 26% gain in FAAM-ADL score and 42% improvement in FAAM-Sport score. Single-leg balance time improved from 3 to 18 seconds with eyes closed (500% increase) and from 22 to 48 seconds with eyes open (118% increase). No ankle sprain episodes occurred during the six-month follow-up period, suggesting the potential preventive efficacy of this approach. While these results are encouraging, the single-case design limits causal inference and generalizability.

Further research through randomized controlled trials with larger cohorts is essential to validate the effectiveness of preventive proprioceptive training in patients with peripheral neurological deficits. If confirmed, systematic integration of proprioceptive assessment and training into the management of L5-S1 radiculopathy could reduce ankle sprain risk in this vulnerable population. This primary prevention approach warrants consideration for inclusion in clinical practice guidelines.
